# Editorial: Self-assembly as a tool for functional materials design

**DOI:** 10.3389/fchem.2022.1010285

**Published:** 2022-09-12

**Authors:** Joanna Pietrasik, Maria Vamvakaki

**Affiliations:** ^1^ Department of Chemistry, Lodz University of Technology, Łódź, Poland; ^2^ Department of Materials Science and Technology, School of Sciences and Engineering, University of Crete, Heraklion, Greece

**Keywords:** self-assembly, functional materials, nano-objects, polymers, soft materials

This Research Topic entitled “*Self-assembly as a tool for functional materials design*” includes an attractive collection of seven (7) manuscripts reporting on the field of soft-matter self-assembly. The articles refer to both small and high molecular weight organic molecules that can organize to form hierarchical structures with novel and unusual properties, demonstrating the dynamic nature of the research area and the potential of the self-assembled systems in Material Science.

In one of the first examples, by Ding et al. a trimethylammonium functionalized cationic water-soluble pillar[5]arene was described, acting as a supramolecular host for the stabilization of indocyanine green dye. The prepared host-guest assemblies enhanced the stability and photothermal conversion efficiency of the photosensitizer and therefore promoted its anticancer activity.

In another interesting study, Tan et al.investigated the assembly of uracil-functionalized boron-dipyrromethenes which are a promising class of optical dyes whose exceptional optical properties are closely related to their supramolecular structures. Computer simulations have elucidated the molecular interactions leading to the supramolecular assemblies, whereas, the nanotribological properties of the assemblies were determined by atomic force microscopy.

In a very novel approach, Sun et al. showed that photoluminescent nanosized quasi-spherical polymeric assemblies prepared by the hydrothermal reaction of polyacrylonitrile have the ability to photo-induce atom transfer radical polymerization catalyzed by very low, parts per million concentrations of a CuII complex with tris(2-pyridylmethyl)amine. The group has used these assemblies to polymerize both acrylate and methacrylate monomers using blue or green light irradiation and have shed light into the mechanistic aspects of the polymerization by linear sweep voltammetry.

Another timely study was reported by Salminen et al. who introduced poly(butyl acrylate)-based materials presenting dual, permanent and dynamic, cross-links. The chemical cross-linker provided shape stability to the networks, whereas, toughening was achieved *via* the dynamic cross-linkers comprising polymerizable salt ions, which presented reversible ionic interactions. The mechanical properties of the materials were shown to be affected by the extent of chemical and dynamic cross-linking, with the former mainly decreasing the network elasticity, and the later enhancing the strength of the material.

An attractive molecular self-assembled system was also reported by Hagar et al. A new class of supramolecular liquid crystal complexes based on complementary acidic and basic molecules were prepared *via* hydrogen-bonding interactions. The phase behavior of the complexes revealed the formation of enantiotropic mesophases, whose type and mesomorphic temperature range was dependent on the length of the alkoxy chains which influenced strongly the molecular interactions.

In a related study, Mohammady et al. focused on novel supramolecular three-ring Schiff base liquid crystalline complexes formed by para-substituted aniline derivatives and para-pyridine carbaldehyde with equimolar quantities of para-alkoxy benzoic acids. The enantiotropic mesophases were both experimentally and theoretically studied and their thermal and optical characteristics were correlated with the size and the polarity of the aromatic substituents.

Finally, this Research Topic includes an enlightening review article by Krieger et al. which discusses the state-of-the-art on electrostatic self-assembly towards the formation of functional nano-objects in solution and compares them against the corresponding solid materials and assemblies prepared by other noncovalent interactions. The combination of electrostatic interactions with additional molecular effects and interactions for the targeted design of versatile structures with a variety of architectures is presented. The system thermodynamics dominating the self-assembly of the building blocks, the formation of stimuli-triggerable structures and specific energy-related applications of the nano-objects are also reported.

